# Supplementary material for: Impaired expression of metallothioneins contributes to allergen-induced inflammation in patients with atopic dermatitis
Source: Nat Commun. 2023 May 19;14:2880. doi: 10.1038/s41467-023-38588-1 (PMC10199008; doi:10.1038/s41467-023-38588-1)
Supplement: Supplementary file 1 — Supplementary Information [file 41467_2023_38588_MOESM1_ESM.pdf]

Supplementary Materials

Impaired expression of metallothioneins contributes to Th17/TNF-mediated, allergen - induced inflammation in patients with atopic dermatitis.

Author List: Sofia Sirvent[1]#, Andres Vallejo[1]#, Emma Corden[2], Ying Teo[1,2], James Davies[1,3], Kalum Clayton[1], Eleanor Seaby[4], Chester Lai[1], Sarah Ennis[4], Rfeef Alyami[1], Gemma Douilhet[1], Lareb S N Dean[1], Matthew Loxham[1], Sarah Horswill[2], Eugene Healy[1], Graham Roberts[2,4], Nigel J. Hall[2,5], Peter S. Friedmann[1], Harinder Singh[6], Clare L. Bennett[3], Michael Ardern-Jones[1,2,7], Marta E Polak[1,7,§,*]

1. Clinical and Experimental Sciences, Faculty of Medicine, University of Southampton; Southampton, UK
2. University Hospital Southampton NHS Foundation Trust, Southampton, UK
3. Department of Haematology, University College London (UCL) Cancer Institute; London WC1E 6DD, UK
4. Human Development and Health, Faculty of Medicine, University of Southampton; Southampton, UK
5. University Surgery Unit, Faculty of Medicine, University of Southampton, Southampton, UK
6. Departments of Immunology and Computational and Systems Biology, The University of Pittsburgh; Pittsburgh, USA
7. Institute for Life Sciences, University of Southampton; Southampton, UK

# These authors contributed equally

*Correspondence to
MEP: m.e.polak@soton.ac.uk
Dr. Marta E Polak
Systems Immunology Group
Clinical and Experimental Sciences
Faculty of Medicine
University of Southampton,
SO16 6YD, Southampton, UK

[§] Current address:
Janssen R&D
1400 McKean Road
Spring House, PA 19477
mpolak1@its.jnj.com

Supplementary Figures

Supplementary Figure 1. In vivo allergen challenge model to investigate mechanisms of local immune responses in human skin.

Supplementary Figure 2 Reactivity to HDM is associated with co-expansion of T cells and LCs.

Supplementary Figure 3 Activated TNF-expressing Th17 cells are significantly enriched in reactive patients.

Supplementary Figure 4 HDM reactivity in reactive patients is mediated by LC : T cell TNF crosstalk and impairs LC transcriptional programming.

Supplementary Figure 5 Enhanced expression of metallothionein genes protects non-reactive patients from inflammation and prevents HDM-induced oxidative stress.

Supplementary Data: attached as csv files

Supplementary Data 1	Patient characteristics
Supplementary Data 2	FLG mutations
Supplementary Data 3	KC signatures
Supplementary Data 4	Panel of genes for Constellation-seq
Supplementary Data 5	Constellation seq analysis: Cluster defining marker genes
Supplementary Data 6	Gene Ontology analysis in DEG from patients responding to HDM
Supplementary Data 7	LC transcriptional programme in control samples
Supplementary Data 8	BioLayout modules and Gene Ontology analysis
Supplementary Data 9	LC signature for Tcell:LC cross-talk
Supplementary Data 10	WGCNA modules and Gene Ontology analysis
Supplementary Data 11	DEGs in KC Constellation-seq
Supplementary Data 12	WGCNA module Turquoise: Top 50 genes
Supplementary Data 13	Gene signatures for GWAS
Supplementary Data 14	Antibodies used in the study

Supplementary Figures

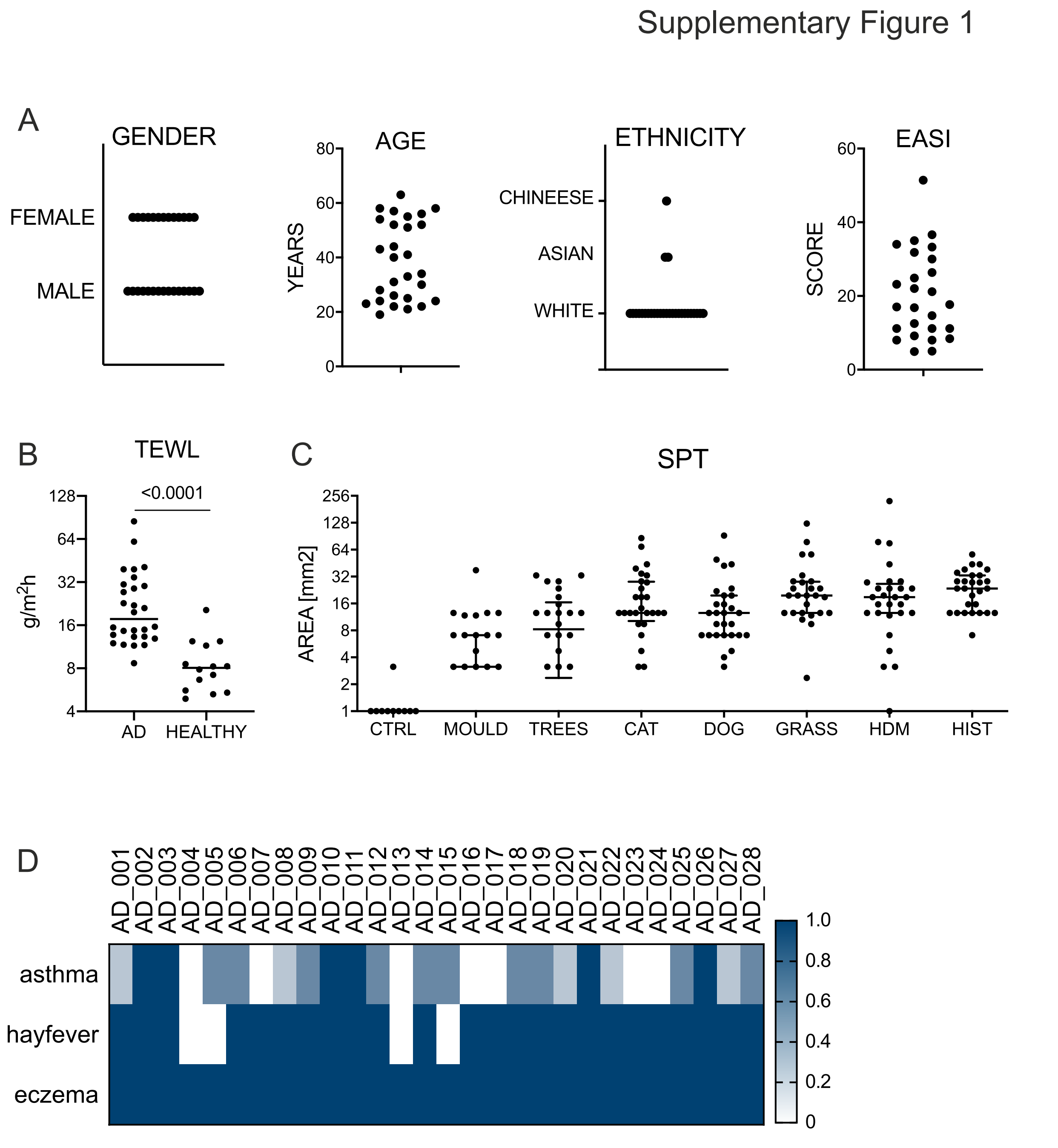

Supplementary Figure 1. *In vivo* allergen challenge model to investigate mechanisms of local immune responses in human skin.
Patient cohort characteristics: A) age, gender, ethnicity, EASI score, n=28. B) trans-epidermal water loss (TEWL) in patients with AD (n=28) and healthy controls (n=14), Mann-Whitney U test, two-sidedC) Skin Prick Test (SPT) responses to stimulation with 6 most common allergens (wheal area given). D) Recorded responses to ISAAC questionnaire, 1 represents 100% of positive responses to questions in a category. Source data are provided as a Source Data file.

Supplementary Figure 2

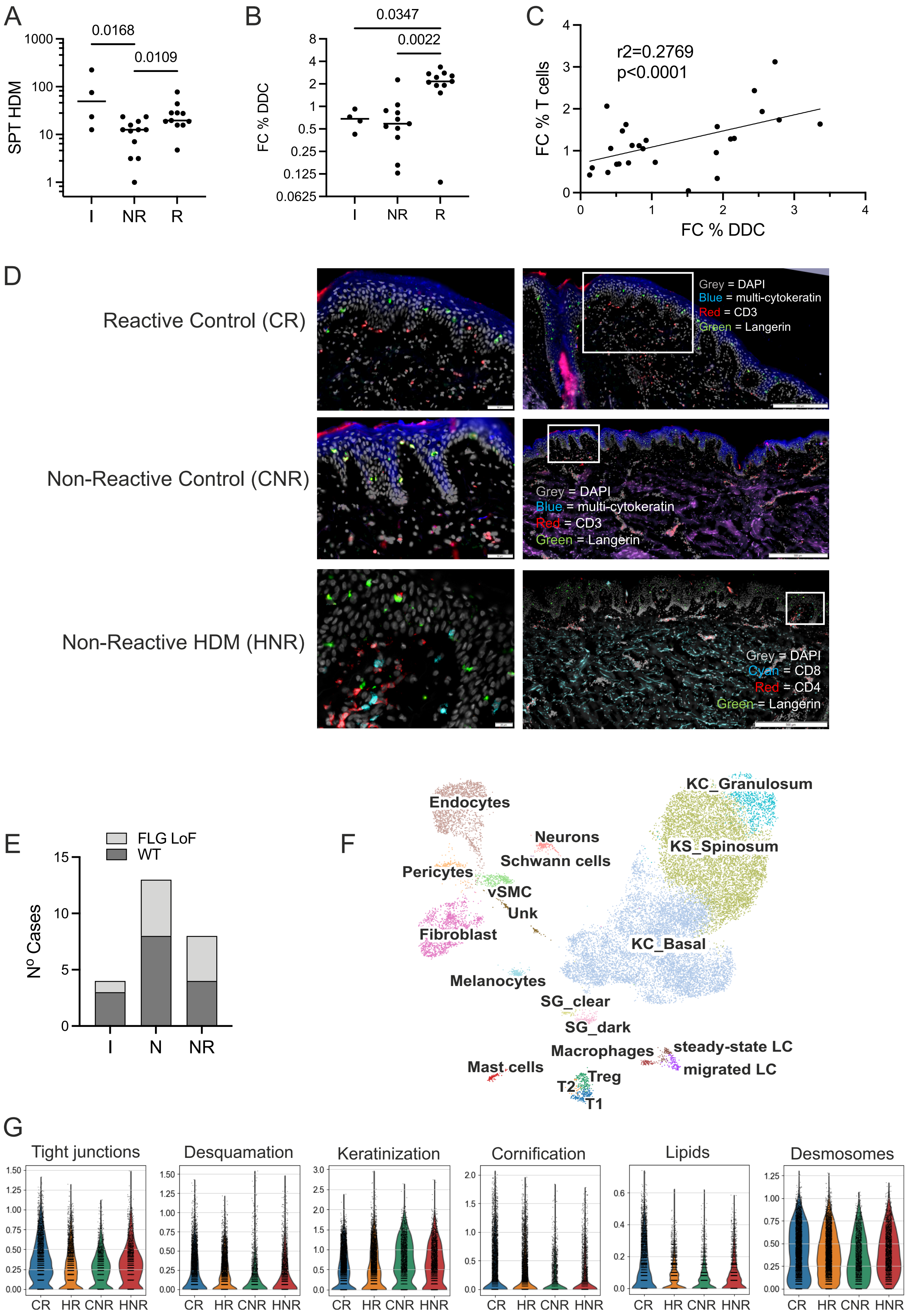

Supplementary Figure 2 Reactivity to HDM is associated with co-expansion of T cells and LCs. A) Skin prick test responses to HDM across patient groups, wheal area mm^2. I n=4, NR n=12, R n=11 Kruskal-Wallis test with post-hoc Dunn test. B) Fold changes in percentage of detected DDC between HDM patch test and control patch test from patients with irritant, non-reactive and reactive reactions to HDM. I n=4, NR n=12, R n=11 Kruskal-Wallis test with post-hoc Dunn test. C) Correlations between fold changes in percentage of CD3+ T cells and DDCs. Pearson correlation coefficient shown for linear regression. 7 SNP measured using Whole Genome Sequencing, n=27 patients. D) Immunofluorescence staining of patch test sites from reactive and non-reactive patients. Inserts show the indicated optical fields. CD207 (green) and CD3 (red). Epidermal layer stained with multi-cytokeratin (blue). DAPI stain for nuclei (grey). Scale bars: 500μm, 50μm, 20μm (inserts). In patch test from HDM exposed non-reactive patient example of interaction between CD4 (red) and CD8 (cyan) T cells shown. E) Number of irritant (I), non-reactive (N), and reactive (NR) cases with loss of function (LoF) variants in FLG compared to wildtype (WT). F) UMAP plot of Drop-seq data, depicting clustering of specific cell populations, n=3 patients, 6 paired samples (Control and HDM) G) Violin plots showing Z score of KC inflammation signature across skin layers, CR: control reactive, HR: HDM reactive, CNR: control non-reactive, HNR: HDM non-reactive. Z scores for keratinocyte gene expression programmes, DropSeq whole transcriptome analysis, fresh tissue, n=6 paired biopsies from 3 donors CR: control reactive, HR: HDM reactive, CNR: control non-reactive, HNR: HDM non-reactive. Source data are provided as a Source Data file.

Supplementary Figure 3

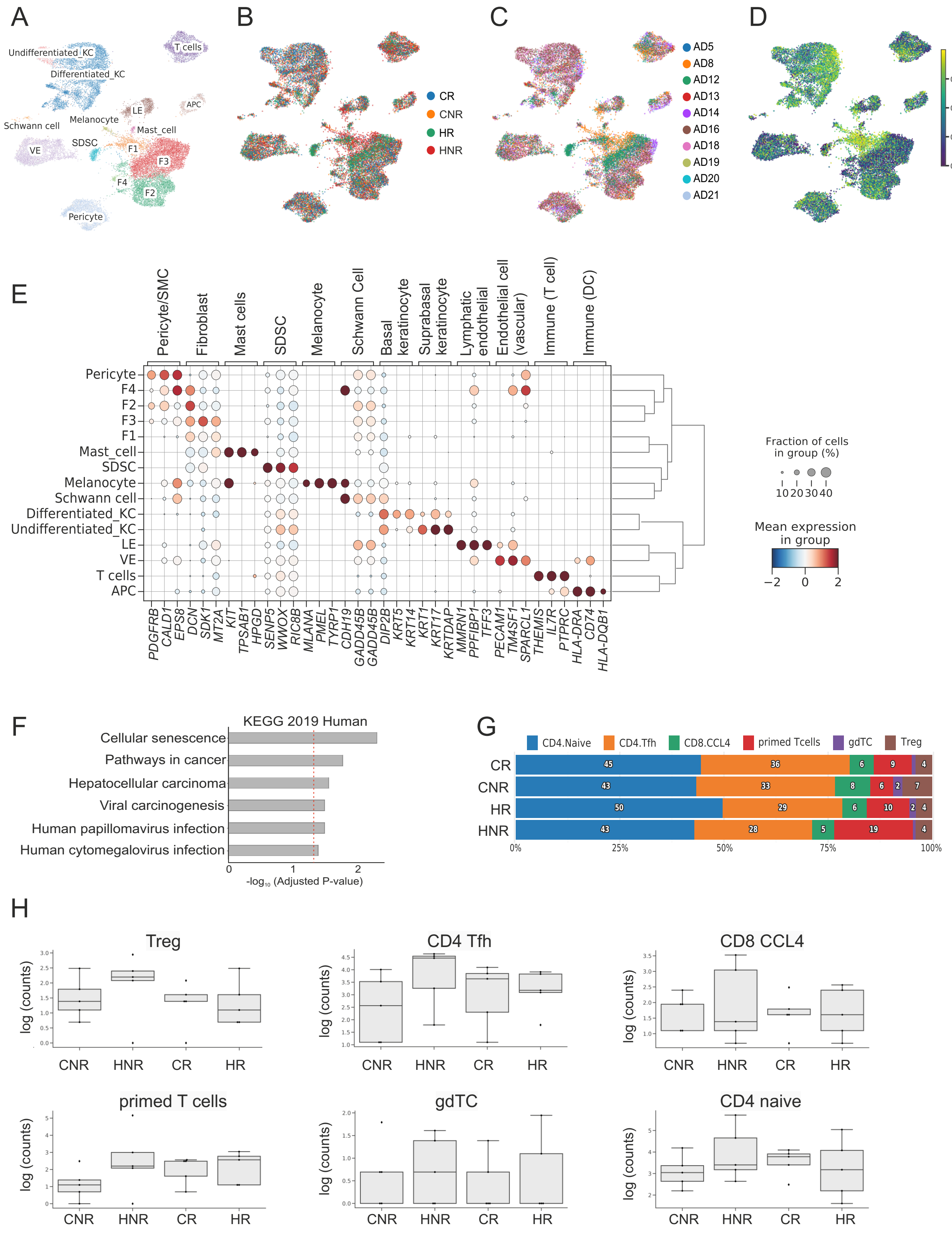

93
94

Supplementary Figure 3

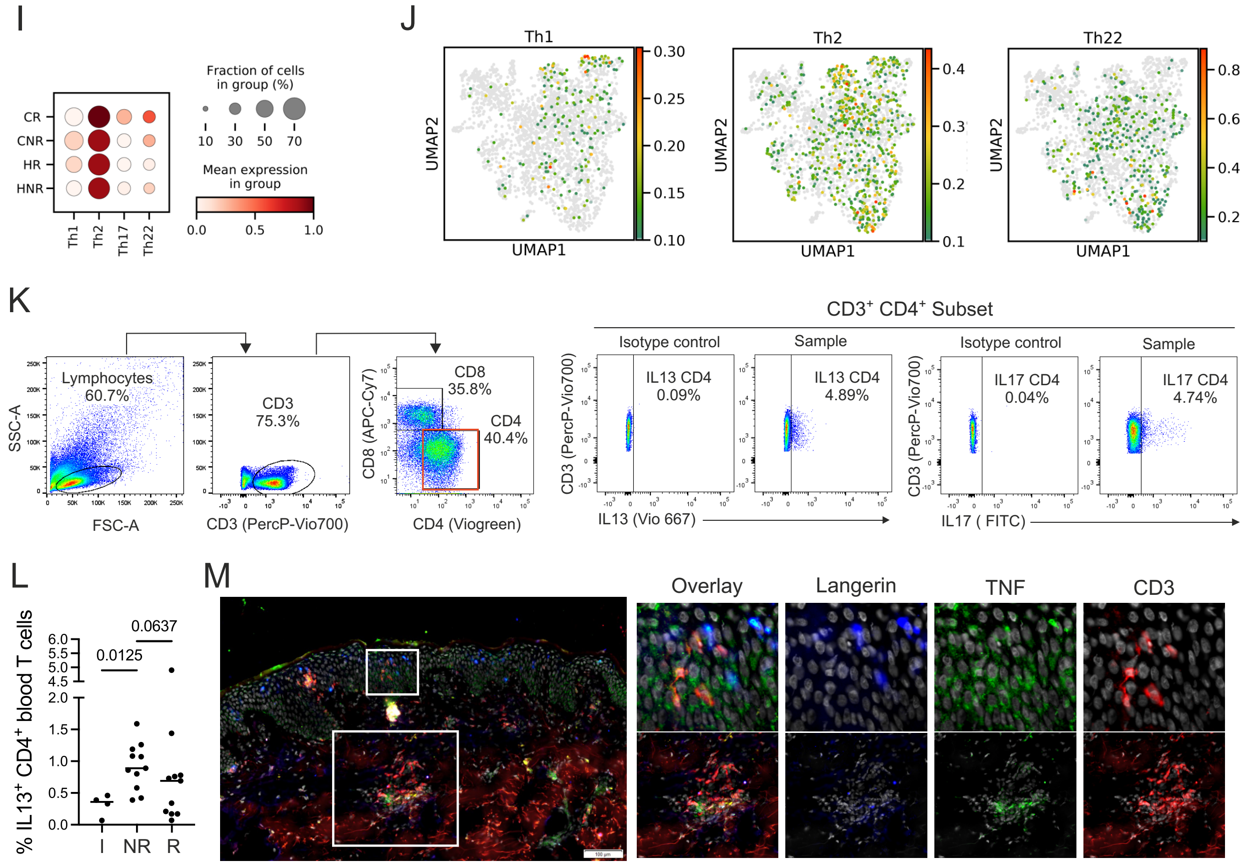

Supplementary Figure 3 Activated TNF-expressing Th17 cells are significantly enriched in reactive patients

A-E) Constellation-seq analysis enriched for 1161 transcripts in 25844 single cells from patch test skin biopsies, n=10 patients A) UMAP plot depicting clustering of specific cell populations B-D) Quality controls for constellation-seq analysis enriched for 1161 transcripts in 25844 single cells from patch test skin biopsies, n=10 patients B) BBKNN integration across treatment and condition C) BBKNN integration across donors D) percentage of mitochondrial genes across samples UMAP plot depicting clustering of specific cell populations E) Cell subset defining markers (Wilcoxon rank test, two sided). F) Top biological pathways enriched at the control site in DEGs from patient non-reactive (CNR) to HDM (KEGG database). P-value computed using the Fisher exact test, with Benjamini Hocheberg FDR correction. G) T cell subset composition across patient groups H) Composition of T cell subsets (HCA profiles) across patch test phenotypes. CR: control reactive, HR: HDM reactive, CNR: control non-reactive, HNR: HDM non-reactive, n=5 individual donors per group, paired. In boxplots, the central line denotes the median, boxes represent the interquartile range (IQR), and whiskers show the distribution except for outliers.I-J) Th immunotypes gene signature across patient groups, I) Th immunophenotype dotplot: size depict % of expressing cells, colour intensity encodes mean expression in group. J) UMAP plot of 2374 single T lymphocytes cells. Constellation-seq analysis enriched for 1161 transcripts from patch test skin biopsies, n=10 patients, 5 per group. Th immunotypes for T cell polarisation shown (Z-score, green to red). K) Gating strategy for flow cytometry analysis of intracellular cytokine staining. L) Expression of IL13 in CD3+CD4+ blood T cells across study groups. Kruskal-Wallis test with post-hoc Dunn test M) Immunofluorescence staining of patch test sites from reactive and non-reactive patients. Inserts show the indicated optical fields. CD207 (blue), CD3 (red) and TNF (green). DAPI stain for nuclei (grey). Overlap

shown in yellow. Scale bars: 100μm. A representative of n=3 individual donors. Source data are provided as a Source Data file.

Supplementary Figure 4

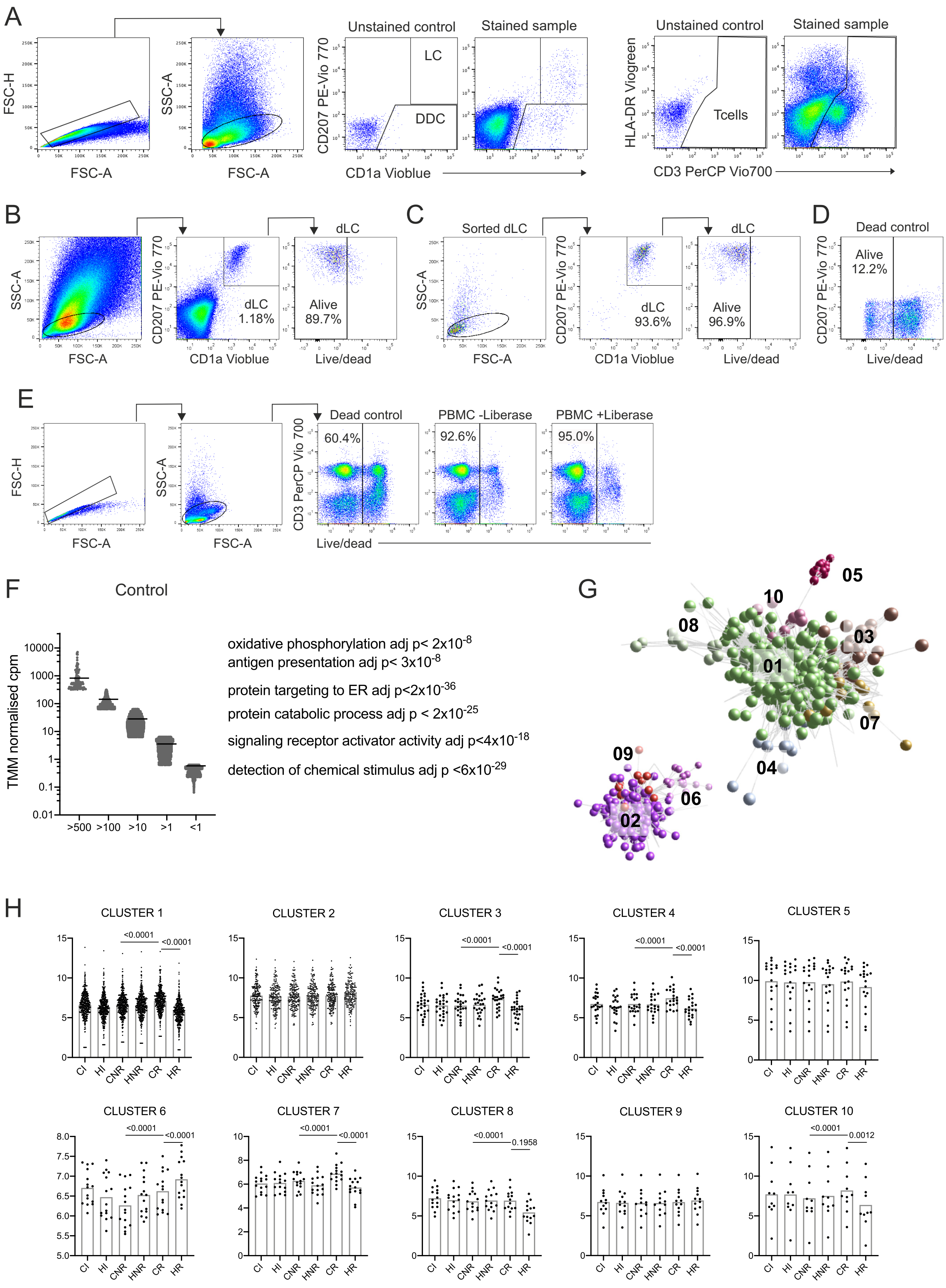

Supplementary Figure 4

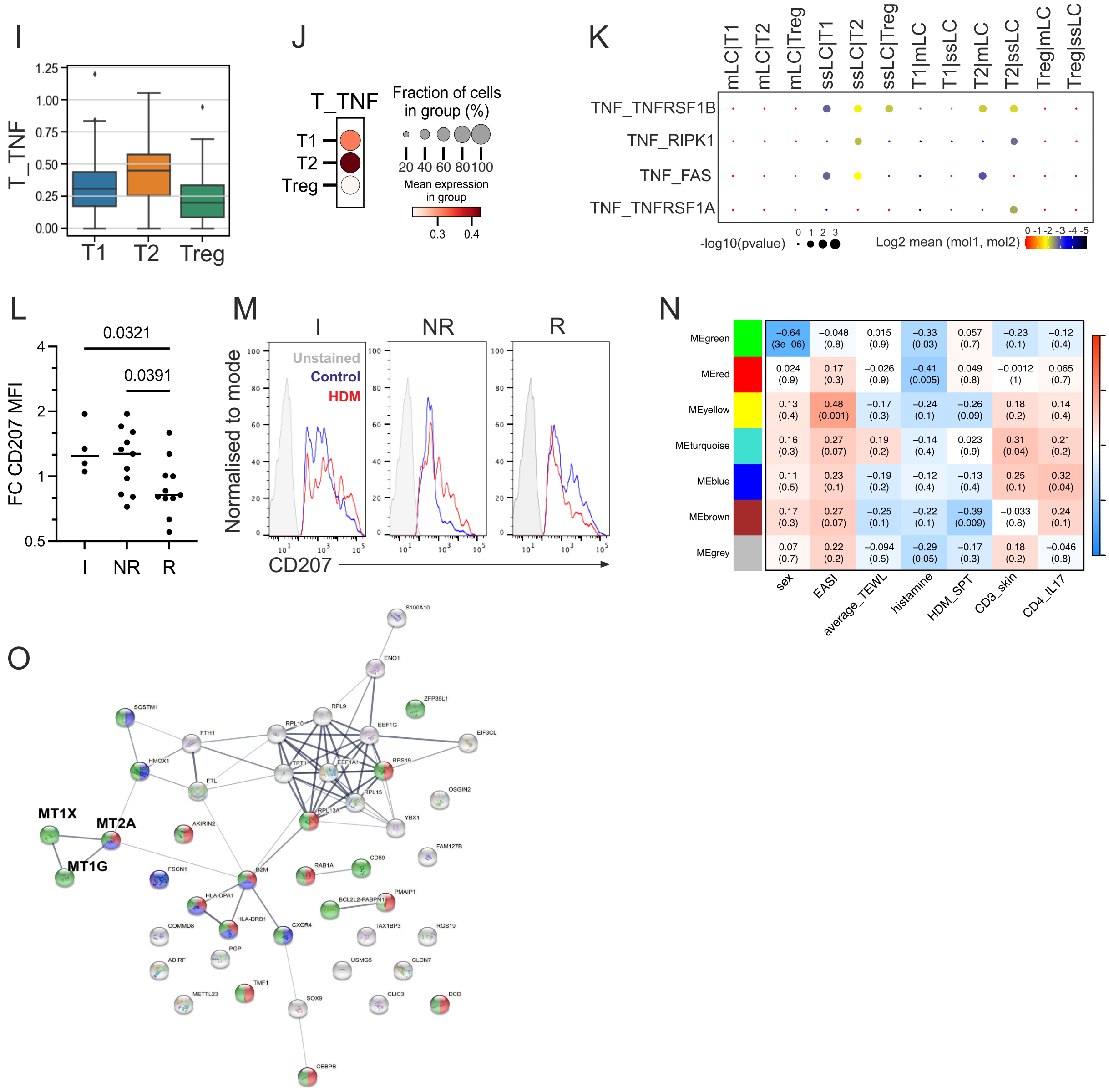

Supplementary Figure 4 HDM reactivity in reactive patients is mediated by LC : T cell TNF crosstalk and impairs LC transcriptional programming

A) Gating strategy for sorting CD207+CD1a+ Langerhans cells, B-D) Viability of LC post digestion procedure prior (B) and post (C) sorting in comparison with dead control (D), E) Viability and preservation of CD3 expression in blood PBMCs post digestion procedure F) Quantitative transcriptional profile of LC in the control skin across all patch tests. Average gene expression binned for the expression level shown. Gene ontology ranked with FDR adjusted p-values given for each gene expression level bracket. TMM-normalised counts. n = 22 independent biological donors (following RNA-seq data QC). ToppGene, Benjamini-Hochberg-adjusted p-value G) Transcript-to-transcript correlation network of 10 largest clusters detected in LC transcriptomes. Lines (edges) represent the similarity between transcripts, circles (nodes) represent genes. H) Mean (± SEM) expression profiles for clusters 1-10, across sample groups. Each dot

represents an average gene expression. CI: control irritant, HI: HDM irritant, CR: control reactive, HR: HDM reactive, CNR: control non-reactive, HNR: HDM non-reactive. Dots represent average expression of transcript. Statistical comparison done using Mann Whitney test for paired samples. I n= 4, NR n = 11, R n=7 (following RNA-seq data QC). I-J) TNF expression across T cell clusters (single cell data, fresh full skin biopsy, n=6 paired biopsies from 3 patients, Drop-seq, I) The center line indicates the median, the edges of the box represent the first and third quartiles, and the whiskers extend to span a 1.5 interquartile range from the edges) K) Dotplot of TNF interactions detected between mature (mLC) and steady state LC (ssLC) and different T cell populations. The size of the dot indicates –log(10) pvalue, as calculated by CellPhone DB package, denoting likelihood of cell-type specificity of a given receptor–ligand. Complex the color denotes log(2) average activation strength. P-value computed using the Fisher exact test, with Benjamini Hocheberg FDR correction.

L) HDM-induced change in CD207 expression levels measured by flow cytometry across patient groups n=27, t-test M) A representative histogram of CD207 expression in I, NR and R patients in control (blue) or HDM-challenged (red) skin, compared with unstained control (grey). Kruskal-Wallis test with post-hoc Dunn test. I n=4, NR n=11, R n=11 N) WGCNA analysis of correlation between clinical and experimental features, recorded as continuous variables and eigenvectors representing modules of co-expressed genes across LC transcriptomes. Each colour coded module (left) represents co-expressed genes. Clinical/experimental features are labelled across X axis. Person correlation coefficients and p-values univariate regression model with pairwise complete Student T test, are given for each correlation, the heatmap (blue to red) indicates correlation strength and direction. O) Protein interaction analysis (STRING) of 50 top genes in LC reversely correlated with CD3 T cell frequency identifies metallothionein functional importance for metal transition metal ion homeostasis (red), cytokine mediated signalling (blue) and responses to stress (green). Source data are provided as a Source Data file.

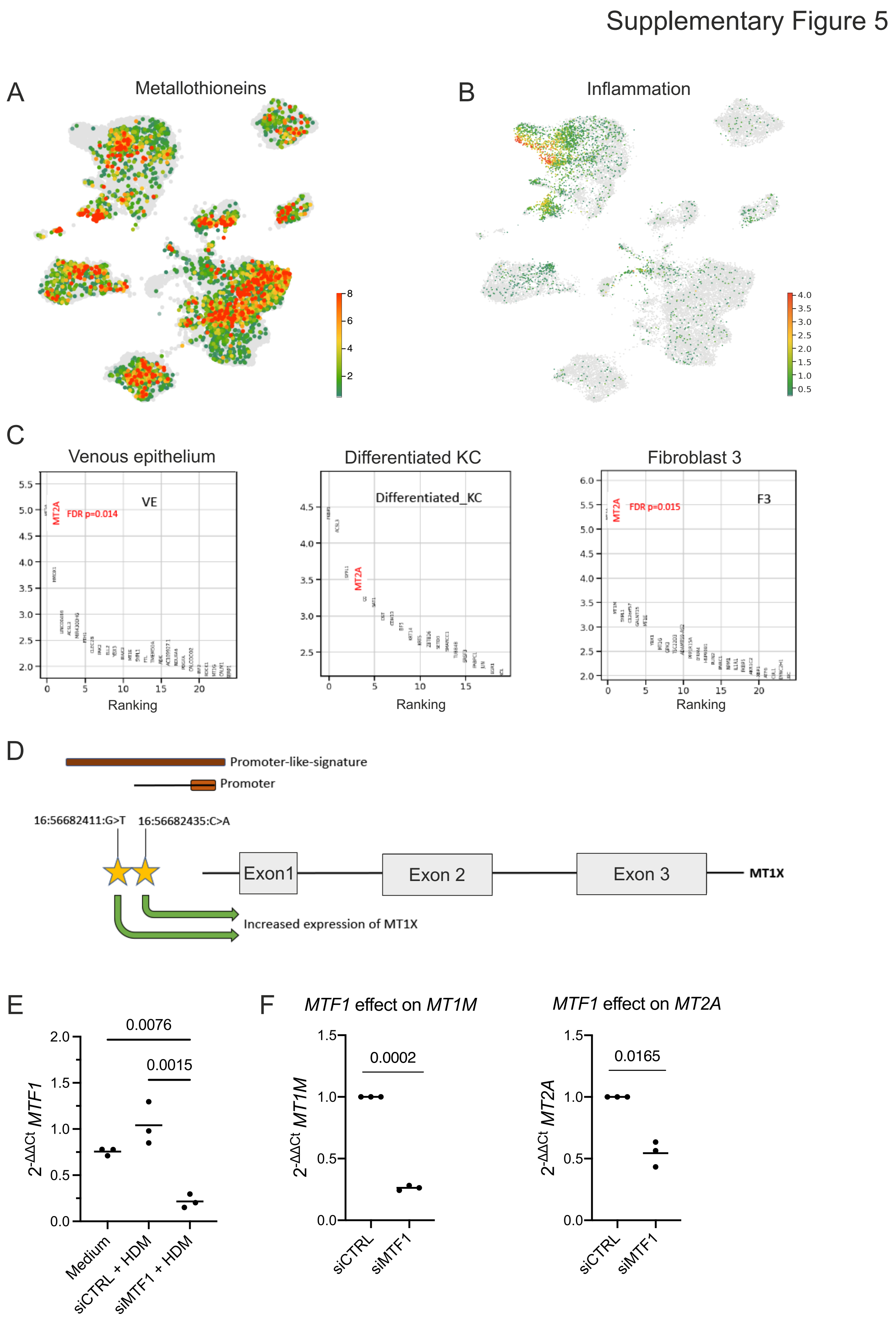
Supplementary Figure 5
A
Metallothioneins
B
Inflammation
C
Venous epithelium
VE
MT2A
FDR p=0.014
Ranking
Differentiated KC
Differentiated_KC
MT2A
Ranking
Fibroblast 3
F3
MT2A
FDR p=0.015
Ranking
D
Promoter-like-signature
Promoter
16:56682411:G>T
16:56682435:C>A
Exon1
Exon 2
Exon 3
MT1X
Increased expression of MT1X
E
0.0076
0.0015
2-ΔΔCt MTF1
Medium
siCTRL + HDM
siMTF1 + HDM
F
MTF1 effect on MT1M
0.0002
2-ΔΔCt MT1M
siCTRL
siMTF1
MTF1 effect on MT2A
0.0165
2-ΔΔCt MT2A
siCTRL
siMTF1

Supplementary Figure 5 Enhanced expression of metallothionein genes protects non-reactive patients from inflammation and prevents HDM-induced oxidative stress

A,C) Constellation-seq analysis enriched for 1161 transcripts in 25844 single cells from patch test skin biopsies, n=10 patients, 5 individual donors/group. Expression of A) metallothionein and B) inflammation signatures. C) Differentially expressed genes between specific cell cluster and all other clusters, MAST, p-value computed Wilcoxon signed rank test, with Bonferoni correction, n=5 individual donors/group. D) Schematic depicting localisation of SNP in promoter/enhancer region upstream of MT1X, significantly enriched in patients tolerant to HDM E-F) Effect of *MTF1* silencing on E) *MTF1* F) *MT1M* and *MT2A* expression in human fibroblasts (n=3 independent experiments, E) paired ANOVA with Tukey test, F) paired t test, two-sided). Source data are provided as a Source Data file.
