# Supplementary material for: Impaired expression of metallothioneins contributes to allergen-induced inflammation in patients with atopic dermatitis
Source: Nat Commun. 2023 May 19;14:2880. doi: 10.1038/s41467-023-38588-1 (PMC10199008; doi:10.1038/s41467-023-38588-1)
Supplement: Supplementary file 3 — Description of Additional Supplementary Files [file 41467_2023_38588_MOESM3_ESM.pdf]

Description of Additional Supplementary Files

Supplementary Data 1 Patient characteristics

Supplementary Data 2 FLG mutations

Supplementary Data 3 KC signatures

Supplementary Data 4 Panel of genes for Constellation-seq

Supplementary Data 5 Constellation seq analysis: Cluster defining marker genes

Supplementary Data 6 Gene Ontology analysis in DEG from patients responding to HDM

Supplementary Data 7 LC transcriptional programme in control samples

Supplementary Data 8 BioLayout modules and Gene Ontology analysis

Supplementary Data 9 LC signature for Tcell:LC cross-talk

Supplementary Data 10 WGCNA modules and Gene Ontology analysis

Supplementary Data 11 DEGs in KC Constellation-seq

Supplementary Data 12 WGCNA module Turquoise: Top 50 genes

Supplementary Data 13 Gene signatures for GWAS

Supplementary Data 14 Antibodies used in the study
