# Supplementary material for: Impaired expression of metallothioneins contributes to allergen-induced inflammation in patients with atopic dermatitis
Source: Nat Commun. 2023 May 19;14:2880. doi: 10.1038/s41467-023-38588-1 (PMC10199008; doi:10.1038/s41467-023-38588-1)
Supplement: Supplementary file 18 — Reporting Summary [file 41467_2023_38588_MOESM18_ESM.pdf]

nature portfolio

Corresponding author(s): Marta E Polak

Last updated by author(s): 04/04/2023

Reporting Summary

Nature Portfolio wishes to improve the reproducibility of the work that we publish. This form provides structure for consistency and transparency in reporting. For further information on Nature Portfolio policies, see our Editorial Policies and the Editorial Policy Checklist.

Statistics

For all statistical analyses, confirm that the following items are present in the figure legend, table legend, main text, or Methods section.

n/a	Confirmed	
☐	☒	The exact sample size (*n*) for each experimental group/condition, given as a discrete number and unit of measurement
☐	☒	A statement on whether measurements were taken from distinct samples or whether the same sample was measured repeatedly
☐	☒	The statistical test(s) used AND whether they are one- or two-sided<br>*Only common tests should be described solely by name; describe more complex techniques in the Methods section.*
☐	☒	A description of all covariates tested
☐	☒	A description of any assumptions or corrections, such as tests of normality and adjustment for multiple comparisons
☐	☒	A full description of the statistical parameters including central tendency (e.g. means) or other basic estimates (e.g. regression coefficient) AND variation (e.g. standard deviation) or associated estimates of uncertainty (e.g. confidence intervals)
☐	☒	For null hypothesis testing, the test statistic (e.g. *F*, *t*, *r*) with confidence intervals, effect sizes, degrees of freedom and *P* value noted<br>*Give P values as exact values whenever suitable.*
☒	☐	For Bayesian analysis, information on the choice of priors and Markov chain Monte Carlo settings
☒	☐	For hierarchical and complex designs, identification of the appropriate level for tests and full reporting of outcomes
☐	☒	Estimates of effect sizes (e.g. Cohen's *d*, Pearson's *r*), indicating how they were calculated

Our web collection on statistics for biologists contains articles on many of the points above.

Software and code

Policy information about availability of computer code

Data collection	The code is available through: https://github.com/afvallejo/AD_2023
Data analysis	All clinical and laboratory statistical analysis were carried out using GraphPad Prism V9.2.0<br>RNA-seq alignment to human genome (GRCh38) and processing was done using Kallisto v0.46.1, Bustools v0.39.3, CellPhoneDB v2.0.0, Scanpy 1.7.1, MAST 1.24.1, CrossTalkeR 1.2.1, Gseapy 0.10.4,<br>WGCNA analysis : 1.71, R v 4.0.3<br>Visualization and clustering of scRNAseq was performed in ScanPy 1.7.1<br>Whole Exome Data Analysis: raw fastq files were aligned to the human genome reference GRCh38 with additional HLA regions included. Alignment was performed using BWA-MEM v0.7.15-r1140 and Samtools 75 v1.3.1. Annotation was completed using Ensembl VEP77 v103, QC metrics data were compiled using R v 4.0.2

For manuscripts utilizing custom algorithms or software that are central to the research but not yet described in published literature, software must be made available to editors and reviewers. We strongly encourage code deposition in a community repository (e.g. GitHub). See the Nature Portfolio guidelines for submitting code & software for further information.

Data

Policy information about availability of data

All manuscripts must include a data availability statement. This statement should provide the following information, where applicable:

- Accession codes, unique identifiers, or web links for publicly available datasets
- A description of any restrictions on data availability
- For clinical datasets or third party data, please ensure that the statement adheres to our policy

Sequencing data for RNA-seq and scRNA-seq is stored in Gene Expression Omnibus database, GSE184509, https://www.ncbi.nlm.nih.gov/geo/query/acc.cgi?acc=GSE18459 . The exome sequencing data underlying this article cannot be shared publicly due to ethical considerations.

Human research participants

Policy information about studies involving human research participants and Sex and Gender in Research.

Reporting on sex and gender	Patient sex was reported follwing self-reporting in ISAAC questionnaire. The study group was biased, with 15 male and 13 female patients.
Population characteristics	On enrolment, information about participants' demographics and previous medical history, immediate family history and information about atopic disease (eg eczema, rhinitis) in the subject was collected based on the ISAAC questionnaire. 89% (25/28) patients were of Caucasian ethnicity, with median age = 37 years, (IQR 24.25-53.50). Eczema severity scores (EASI) indicated moderate to severe disease (median = 17.7, IQR:10.2 — 30.9, max = 51.4, Supplementary Figure 1A, Supplementary Table 1). Skin barrier was measured as transepidermal water loss (TEWL) of non-eczemtaous sites and was impaired in the AD patients: median = 17.7 g/m2h, IQR:13.3 — 30.7, max = 85.0 compared to healthy; median = 8.1 g/m2h, IQR = 5.9-10.8, p<0.0001).
Recruitment	Adult AD patients with mild to severe disease (mean objective EASI) were recruited through the Dermatology Centre, Southampton University Hospital NHS Trust. The patients were recruited from a specialist atopic dermatitis clinic and were therefore selected as moderate severe cases. All patients eligible for the study (i.e. meeting moderate to severe AD criteria) were invited, and had a choice to participate. No recruitment biased was noted.
Ethics oversight	Informed, written consent was obtained as per approval South East Coast - Brighton & Sussex Research Ethics Committee in adherence to Helsinki Guidelines (approval: 16/LO/0999)

Note that full information on the approval of the study protocol must also be provided in the manuscript.

Field-specific reporting

Please select the one below that is the best fit for your research. If you are not sure, read the appropriate sections before making your selection.

[x] Life sciences   [ ] Behavioural & social sciences   [ ] Ecological, evolutionary & environmental sciences

For a reference copy of the document with all sections, see nature.com/documents/nr-reporting-summary-flat.pdf

Life sciences study design

All studies must disclose on these points even when the disclosure is negative.

Sample size	Power calculations for RNA-seq experiment, which was the most limiting for the study, were done using "RNASeqPower" package in Bioconductor, R, based on the preliminary data measuring expression levels of key molecular hubs in the LC gene regulatory network after exposure to epidermal cytokines. To detect a statistically significant effect in a randomized case-control experiment with 20 million reads sequencing depth, experimental variation cv=0.5, α=0.01, 11biological replicates per group provide >8S% power to detect a 2 fold difference in gene expression levels. To detect a statistically significant difference of two-fold difference in a case-control matched perturbation experiment/time series (α=0.05, power>80%), sample size of 3 is sufficient.
Data exclusions	28 patients were recruited. One of the samples has been excluded due to disagreement on the PT outcome (observed minimal redness, but not consistent with the patch test perimeter). FACS results for CD3 T cells were compromised for one sample due to a technical fault. One sample has been processed for single cell RNA-seq only. Additional QC inclusion criteria were applied for bioinformatic analysis (final n=22).
Replication	Cell composition has been assessed using Flow Cytometry (54 samples, 27 patients), scRNA-sequencing (20 samples, 10 patients, 5 per group) and additionally tested using immunofluorescence (6 samples, 3 patients). Importance of identified regulators were confirmed between skin and blood (27 patients), in GWAS study (27 patients) and in functinal experiments (cell line, 3 independent experiments). No data was excluded (following quality checks described above).

Randomization	The 28 patients were recruited to the study accordingly to the study protocol, specifying active moderate to severe disease. Otherwise, there was no bias in the patient recruitment. Patients were stratified as "irritant" "non-responder" or "responder" depending on the clinical-assessed outcome of 48 patch test with House Dust Mite allergen.
Blinding	The investigators were not blinded on the recruitment or for the analysis. The reported study was not assessing any interventions in patients. The study was designed to test a hypothesis that differences exist in immune cell function in AD patients responding and not responding to topical application of an allergen. The patients were not randomised to study groups, but contrasted based on the clinically detectable response to house dust mite patch. All patients in the study underwent the same procedure. As the clinical response to HDM classified patient group, blinding was not possible.

Reporting for specific materials, systems and methods

We require information from authors about some types of materials, experimental systems and methods used in many studies. Here, indicate whether each material, system or method listed is relevant to your study. If you are not sure if a list item applies to your research, read the appropriate section before selecting a response.

Materials & experimental systems

n/a	Involved in the study	
☐	☒	Antibodies
☐	☒	Eukaryotic cell lines
☒	☐	Palaeontology and archaeology
☒	☐	Animals and other organisms
☒	☐	Clinical data
☒	☐	Dual use research of concern

Methods

n/a	Involved in the study	
☒	☐	ChIP-seq
☐	☒	Flow cytometry
☒	☐	MRI-based neuroimaging

Antibodies

Antibodies used

Full data on antibodies is available in supplementary Table 14.

Antibodies used:

Flow Cytometry: all antibodies designed and validated for Flow Cytometry. All antibodies specifically reactive for human tissues, no cross-reactivity reported by manufacturer
CD1a, CD207:Miltenyi Biotech, UK and HLA-DR: BD Biosciences, UK CD3, CD2S and CD103 (Miltenyi Biotech).
CD3- PerCP S.5 (eBiosciences), CD4-VioGreen, IL13-APC and IL17-FITC (Miltenyi Biotech)

Immunofluorescence: All antibodies designed and validated for immunohistochemistry and/or immunofluorescence
Primary unconjugated antibodies:
CD207, multi-cytokeratin (Leica), reacts with human tissues
CD3 (Dako), validated in Wester blot on T cell cell lines, reacts with human tissues
CD4, CD8, FOXP3, IL17 (Abcam) react with human tissues

Secondary antibodies Alexa Fluor 488 goat anti-mouse IgG1a, Alexa Fluor SSS goat anti-rabbit IgG, and Alexa Fluor 647 goat anti-mouse IgG2b (all from ThermoFisher Scientific).

TotalseqA antibody

Hashtag 1 Antibody TotalSeq fM -A0251 anti-human, L NH-94• 2M2, 394601
Hashtag 2 Antibody TotalSeq r"-A0252 anti-human LNH-94• 2M2, 394603
Hashtag 3 Antibody TotalSeq TM-A0253 anti-human LNH-94• 2M2, 394605
Hashtag 4 Antibody TotalSeq TM -A0254 anti-human LNH-94• 2M2, 394607
Hashtag 5 Antibody TotalSeq fM -A0255 anti-human LNH-94• 2M2, 394609
Hashtag 6 Antibody TotalSeq TM -A0256 anti-human LNH-94• 2M2, 394611
Hashtag 7 Antibody TotalSeq TM -A0257 anti-human LNH-94• 2M2, 394613
Hashtag 8 Antibody TotalSeq TM -A0258 anti-human LNH-94• 2M2, 394615

Validation	All antibodies were used at pre-titrated concentration. The specificity was detailed in the antibody datasheet. Appropriate isotype controls were used to assess the baseline fluorescence.<br><br>Each lot of the antibody is quality control tested by immunofluorescent staining with flow cytometric analysis and the oligomer sequence is confirmed by sequencing. TotalSeq™-A antibodies are compatible with 10x Genomics Single Cell Gene Expression Solutions.

Eukaryotic cell lines

Policy information about cell lines and Sex and Gender in Research

Cell line source(s)	MRC lung fiborblasts were obtained from the European Collection of Authenticated Cell Cultures (ECACC)
Authentication	The cell lines used have not been authenticated
Mycoplasma contamination	All cultures were tested and free of Mycoplasma contamination
Commonly misidentified lines (See ICLAC register)	No commonly misidentified cell lines were used

Flow Cytometry

Plots

Confirm that:

- [x] The axis labels state the marker and fluorochrome used (e.g. CD4-FITC).
- [x] The axis scales are clearly visible. Include numbers along axes only for bottom left plot of group (a 'group' is an analysis of identical markers).
- [x] All plots are contour plots with outliers or pseudocolor plots.
- [x] A numerical value for number of cells or percentage (with statistics) is provided.

Methodology

Sample preparation	All samples analysed were human primary cells. 6 mm biopsies were minced using a surgical scalpel and digested for 16h at 37C with agitation in RPMI with LiberaseTM (Roche) following manufacturer's instructions. After 16h of digestion cells were collected and washed with RPMI S% FBS. Cells were resuspended in PBS 1% BSA 20mM EDTA and filtered through 70um sterile filters before surface antibody staining for FACS. All antibodies were used at pre-titrated, optimal concentrations. For surface staining of live cells buffer containing PBS 1% BSA was used for all antibody staining. For intracellular cytokines freshly isolated PBMCs were activated with anti-CD3 and anti-CD28 (1 mg/ 1), with GolgiPlug (BD Biosciences, Oxford, UK). The Cytofix/Cytoperm kit (BD Biosciences) was used according to the manufacturers' instructions.
Instrument	FACS ARIA Flow Cytometer (BD Biosciences)
Software	FlowJo software (Tree Star, Ashland), V10
Cell population abundance	LC constituted 0.12 - 1.2% , mean 0.44% +- 0.2S% in control skin. CD3+ T cells constituted 4- 29% , mean 14% +- 6.4% in control skin.
Gating strategy	Singlets (FCSA:FCSH), cells (FCS:SSC), CD207+/CD1a+ or HLA-DR/CD3. For intracellular cytokines: CD3- PerCP 5.5 (eBiosciences), CD4-VioGreen (Miltenyi Biotech) followe by IL13-APC and IL-17-FITC versus isotype negative controls

- [x] Tick this box to confirm that a figure exemplifying the gating strategy is provided in the Supplementary Information.
